# Heating Conditions Influence on Solidification of Inconel 625–WC System for Additive Manufacturing

**DOI:** 10.3390/ma13132932

**Published:** 2020-06-30

**Authors:** Jan Huebner, Paweł Rutkowski, Aleksandra Dębowska, Dariusz Kata

**Affiliations:** 1 Faculty of Materials Science and Ceramics, Department of Ceramics and Refractories, AGH University of Science and Technology, al. Mickiewicza 30, 30-059 Krakow, Poland; pawelr@agh.edu.pl (P.R.); kata@agh.edu.pl (D.K.); 2 Faculty of Metals Engineering and Industrial Computer Science, Department of Surface Engineering and Materials Characterisation, AGH University of Science and Technology, al. Mickiewicza 30, 30-059 Krakow, Poland; debovska@agh.edu.pl

**Keywords:** Inconel 625, tungsten carbide, differential thermal analysis, microstructural changes, additive manufacturing, laser processing, Inconel 625–WC, metal matrix composites

## Abstract

In this study, an Inconel 625–tungsten carbide (WC) composite system was investigated by means of microstructure changes affected by both heating rate and WC content. In order to investigate how the system behaves while exposed to fast thermal processing, controlled melting using a differential thermal analysis (DTA) apparatus was performed on the powders. Two WC powders with different average grain size were used to obtain six compositions of Inconel 625–WC powder mixtures (10, 20, and 30 wt.% WC). They were analyzed under 10 and 30 °C/min heating rate in order to obtain composite samples. Results from DTA together with SEM/energy-dispersive X-ray spectroscopy (EDS) microstructural observations allowed observing material changes during solidification. Because of the extensive microsegregation of alloying elements to liquid and their reactions with C, which derived from dissolved WC, the formation of secondary phases with improved microhardness was possible. The collected results provide a better understanding of material behavior during intensive thermal processing which can be useful when designing materials for the laser additive manufacturing technique.

## 1. Introduction

Additive manufacturing (AM) which is sometimes called three-dimensional (3D) printing is constantly and rapidly expanding. With biomedical, aerospace, and power generation applications, 3D printing is widely used across whole spectrum of industrial applications. Because of its relatively low cost, universal applicability, and potential to reduce loss of raw material during the manufacturing process, it started to be recognized for its relative eco-friendliness, in comparison to conventionally used production methods, especially for metal-based materials [1,2,3,4]. Despite the many advantages of AM methods, they met many material- and process optimization-based problems, due to strict requirements which are required for specialized applications. One of the more interesting and perspective uses of 3D printing is the production of turbine blades needed in jet engines. This type of engine is known for its relatively small size, quick start-up, and low emission of exhaust gases. Materials used for such applications need to withstand aggressive working conditions in temperature ups to 1400 °C. However, a cost-efficient way to produce or repair turbines that work in lower temperatures of about 600–650 °C is appealing to global companies like ABB, Siemens, or Westinghouse.

Research on materials that can be used in AM of turbine engine parts is widely developed and discussed. Most reports focus on either nickel- or cobalt-based alloys, which are characterized by good corrosion resistance, excellent weldability, and thermal and chemical stability due to the high content of Cr and Mo. Superalloys like Inconel 625, 718, 738, and 786 (Ni-based) and Stellite 6, 12, and 21 (Co-based) are well known and liked for the above-mentioned properties [5,6,7,8,9]. Inconel 625 is extensively used in applications up to 650 °C due to its chemical composition (presented in Table 1). It is classified as a solid solution-strengthened Ni-based alloy. It exhibits good corrosion resistance in many environments due to the passive layer of Cr and Mo oxides on its surface. The solubility of main alloying elements like Cr and Mo are much higher in comparison to Fe-based alloys. Because of similar atomic size, Cr, Mo, and W are substitutional to Ni; thus, this allows for solid solution strengthening. The addition of these elements improves corrosion resistance and/or changes to how materials behave during welding. Inconel 625 shows increased strength in comparison to commercially pure Nickel alloys [10].

Metallic materials have their limitations like relatively low wear resistance and hardness. These can be overcome by the introduction of ceramic particles into the system. Due to its excellent weldability by both liquid Ni and Co and high hardness, tungsten carbide (WC) can be successfully used as reinforcement. Despite extensive research on the WC addition effect on Ni- and Co-based alloy behavior in laser additive manufacturing [11,12,13,14,15], there are no complex reports focused on material behavior during thermal processing with different conditions. The general simplified behavior of the Inconel 625–WC system during heating should proceed as follows:

(1) $$Powders\;\overset{T↑}{\to }Inconel\;625\;start\;to\;melt\overset{T↑}{\to }WC\;partially\;dissolves\overset{T↑}{\to }liquid\;metal,$$

which is then followed by cooling,

(2) $$Liquid\;metal\overset{T↓}{\to }solidification\;starts\overset{T↓}{\to }\;precipitation\;ofsecondary\;phases\;\overset{T↓}{\to }solidification.$$

**Figure 1:**
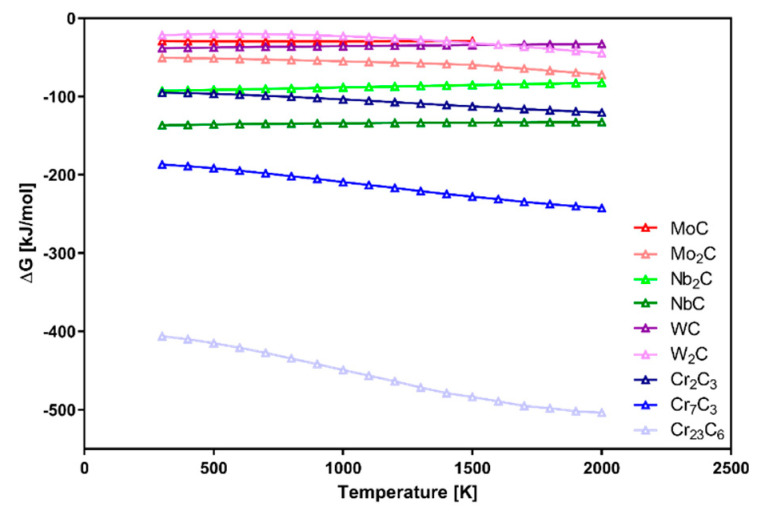
Gibbs free energy for simple carbides that can form in Inconel 625–WC system [16,17,18,19,20,21,22,23,24,25,26].

This shows that materials in the Inconel 625–WC system could include different types of structures: Ni-based matrix, partially dissolved WC during heating, secondary carbides, and γ/intermetallic eutectic structures formed during solidification. Based on research data [16,17,18,19,20,21,22,23,24,25,26] on Gibbs free energy (ΔG), shown in Figure 1, Cr carbides are the easiest to be formed. Because the presented data were measured for simple systems without consideration of possible interferences, the graph can be used as a formation order indicator. In more complicated systems like Inconel 625–WC, alloying elements are the major factor with an influence on the Gibbs free energy of possible phases. Their presence affects the reactions between them and carbon, as well as microsegregation during solidification. The high heating rate typical for laser processing should be considered as an important factor with a significant microstructure refinement effect which modifies the final properties of the manufactured material.

In order to better understand the behavior of Ni-based alloys while WC is added into the system, controlled melting of Inconel 625–WC powder mixtures is proposed in this study. Due to extreme conditions of laser processing (mainly high heating and cooling rates), the experiment was designed to check how the material behaves during exposition to rapid temperature changes. Laser additive manufacturing (LAM) usually uses different types laser sources which can generate a high-energy laser beam which delivers very small areas, leading to extreme temperature increases of about 800–1000 °C/s within the material. Such conditions are difficult to imitate in precise analysis apparatus because it can possibly lead to deceitful results due to the speed of the process. In order to investigate the Inconel 625–WC system behavior during the heating–cooling process, the experiment was done using a differential thermal analysis (DTA) apparatus which allows for very accurate control of temperature during the melting of powders. Powder samples were subjected to two different heating rates of 10 °C/min and 30 °C/min. After cooling, microstructural observations and elemental analysis of the obtained samples revealed how a different content of carbide and a changing heating rate affect the formation of secondary phases in the material. Finally, the average microhardness of different phases was measured to check differences in phases detected in composite material.

## 2. Materials and Methods

The analysis of Inconel 625–WC composite obtained by laser cladding proved that it is a promising method of production for this type of material. Because of the high complexity of the system, analysis of its microstructure and phase composition was challenging. Two different commercially available WC powders (H. C. Starck) were selected and subjected to grain size analysis via a laser diffraction method using Malvern Panalytical Mastersizer 2000 apparatus (Malvern, Worcestershire, UK). The measured average grain size for WC_1 powder was D_WC_1_ = 3.88 µm and that for WC_2 powder was D_WC_2_ = 6.13 µm (Table 2). Mixtures with the addition of WC_1 and WC_2 powder mixtures were prepared by mixing 10, 20, and 30 wt.% WC with Inconel 625 powder with an average size of spherical grains of D_Inc_ = 104 µm.

**Figure 2:**
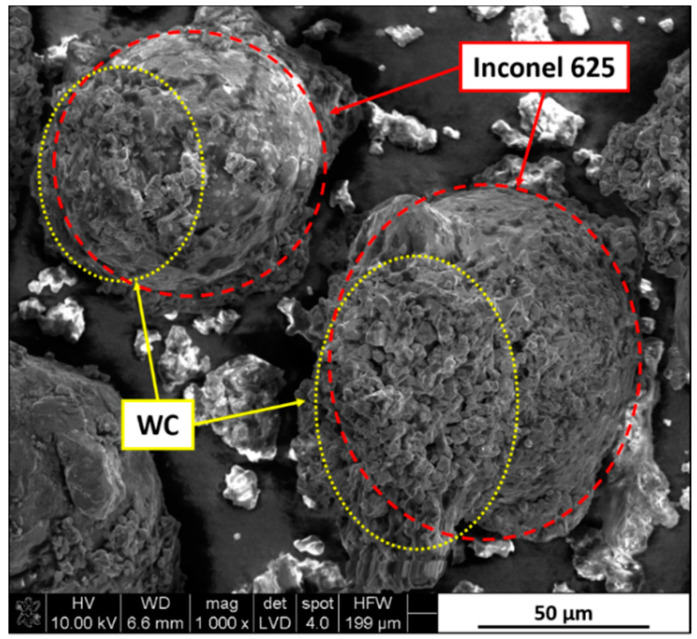
Spherical Inconel 625 particle surface covered by agglomerates of fine tungsten carbide (WC) grains with dextrin binder.

Powders were put in the milling chamber with 0.25 wt.% dextrin binder in the form of a water solution with cemented carbide grinding media in a weight ratio of 1:1. Mixtures were homogenized for 90 min and then sieved and dried. Obtained powders were characterized by a good connection between Inconel 625 and WC grains, as seen in Figure 2.

In order to better understand how the material behaves while exposed to different heating conditions, powder mixtures were subjected to differential thermal analysis (DTA) using a Netzsch STA 449 F3 apparatus (Selb, Bavaria, Germany). This allowed investigating how heating rate affected transformation of the microstructure. A set amount of 2 g of each obtained powder mixture was put inside Al_2_O_3_ crucibles and then individually analyzed in temperatures up to 1450 °C under different heating conditions in argon flow. After reaching maximum temperature, samples were rapidly cooled (cooling rate of about 200 °C/min) to a room temperature of 20 °C. This allowed rapid solidification of obtained samples. The list of prepared samples is presented in Table 3.

In order to prepare samples for SEM/energy-dispersive X-ray spectroscopy (EDS) examination, all of them were ground and polished using a semi-automatic Struers TegraPol-21 polisher (Copenhagen, Hovedstaden, Denmark) equipped with a TegraForce-5 holder.

SEM/EDS point analysis was performed using an FEI Nova NanoSEM 200 (Hillsboro, OR, USA) equipped with an EDAX EDS analyzer. Additionally, microhardness tests were done using a Future-Tech FM-700 hardness tester (Kawasaki, Kanagawa, Japan), in order to investigate differences between phases present in the composite.

## 3. Results

**Figure 3:**
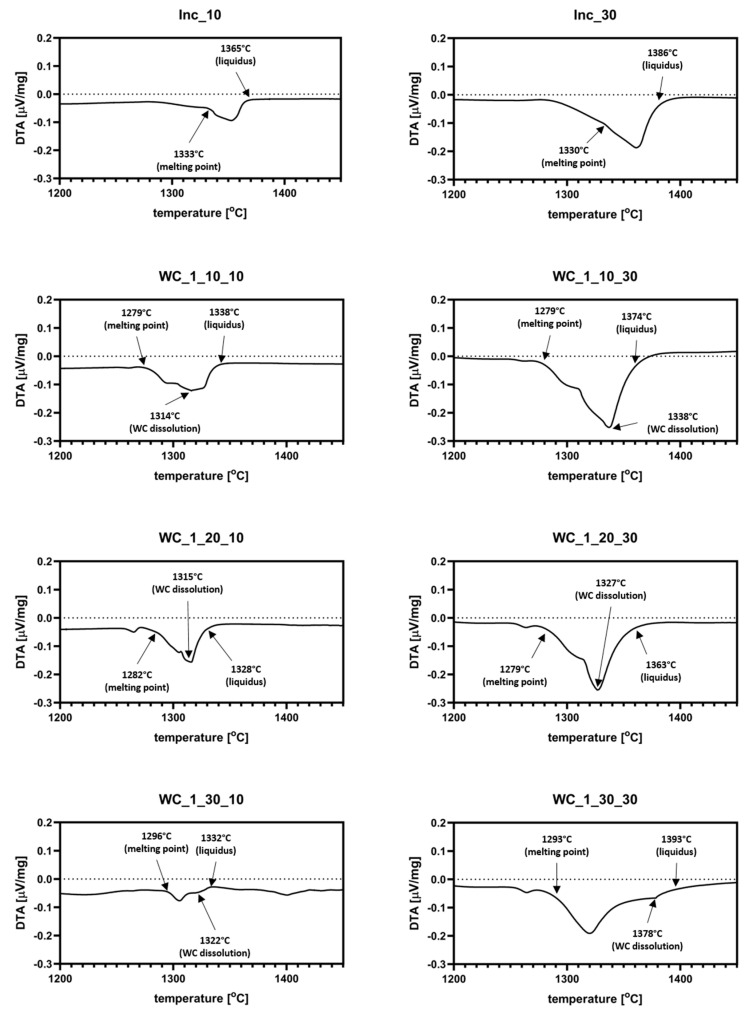
DTA thermograms for Inconel 625–WC_1 samples for 10 °C/min and 30 °C/min heating rates; the first thermal peak is derived from Inconel 625 melting, while the second one represents partial dissolution of WC particles in liquid metal.

**Figure 4:**
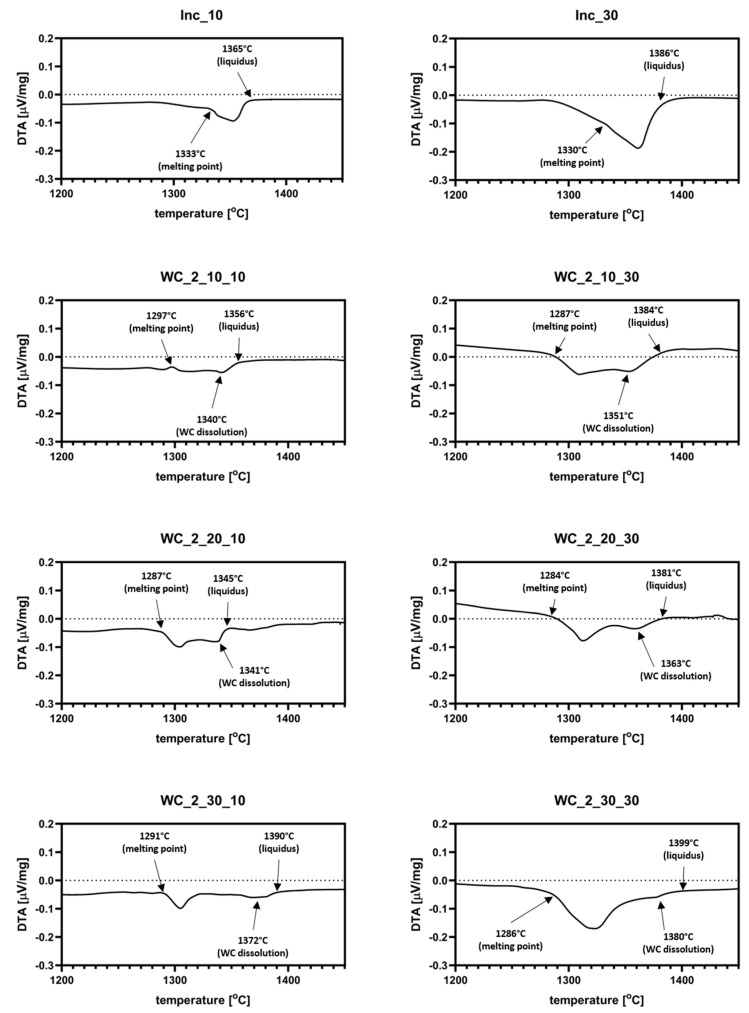
DTA thermograms for Inconel 625–WC_2 samples for 10 °C/min and 30 °C/min heating rates; the first thermal peak is derived from Inconel 625 melting, while the second one represents partial dissolution of WC particles in liquid metal.

The results of DTA analysis are presented in Figure 3 and Figure 4. Additional graphs with datasets for Inconel 625 reference samples are included in both figures. As it can be observed, the pure Inconel 625 sample started melting at about 1330–1333 °C. The addition of WC_1 powder to the system decreased the melting points of materials to about 1263 °C. Most of the samples show two endothermic signals. The first one is derived from melting of Inconel 625. Because of that, liquid metal started surrounding the WC particles, characterized by a good wetting angle, with liquid Ni (about 20°). The second signal indicates the partial dissolution of WC in liquid, which enriched it with both W and C. As shown in Figure 3, the presence of a dextrin binder resulted in a small thermal effect just before the materials reached their respective melting points. This was most likely a result of the carbon–oxygen reaction, which led to its partial evaporation from samples in form of CO_2_. Analysis of samples with the addition of WC_2 powder revealed a similar behavior, as seen in Figure 4. The recorded thermal effect for those materials was noticeably stronger than for WC_1 system, which was the result of the higher heat absorption by coarser WC grains. In samples with 10 and 20 wt.% addition of WC_1 and WC_2 powders, partial dissolution of the WC particles started before reaching maximum reaction speed. This was different for samples with the addition of 30 wt.% WC, where WC dissolution occurred after the reaction speed reached its maximum. The coarser WC_2 particles had less influence on the melting points of mixtures in comparison to pure Inconel 625, as seen in Figure 4. For 20 and 30 wt.% WC_2 samples, this decreased to about 1290 °C. The dissolution of carbide reinforcement was limited because of the increased grain size of WC_2 powder. This resulted in a weaker endothermic signal, as seen in Figure 4. Because data obtained from analysis with a higher heating rate (30 °C/min) resulted in a smoother curve of datasets, due to the smaller density of measurements, determination of the exact temperatures during heating was not as accurate as for the 10 °C/min samples.

**Figure 5:**
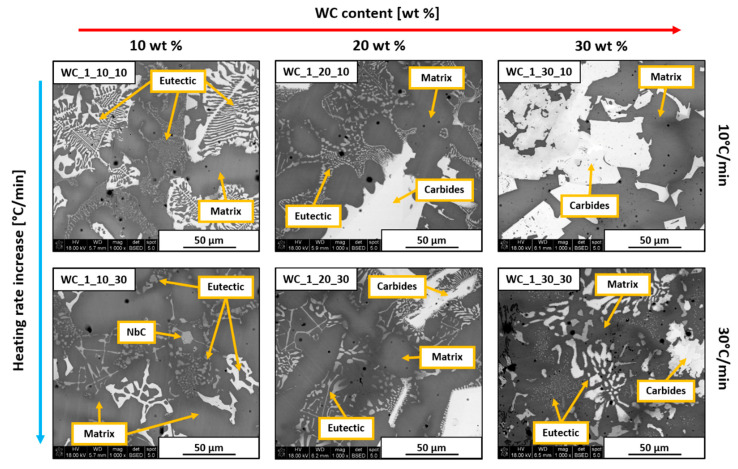
SEM images of Inconel 625–WC_1 sample cross-sections.

**Figure 6:**
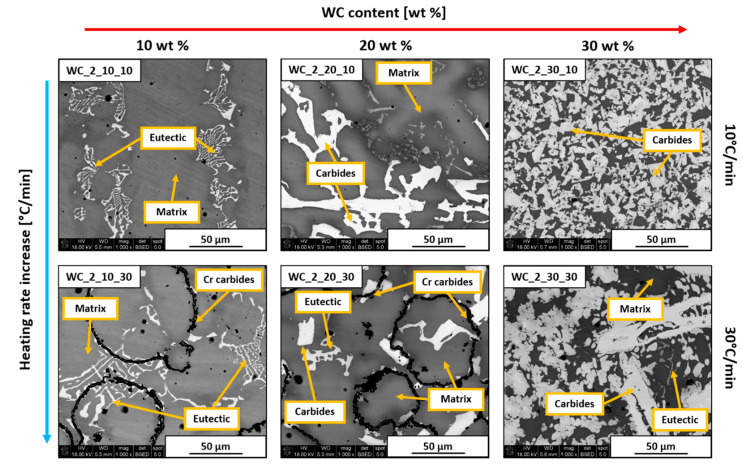
SEM images of Inconel 625–WC_2 sample cross-sections.

SEM images presenting all prepared Inconel 625–WC samples are shown in Figure 5 and Figure 6. All examined materials revealed some amount of porosity in the form of small circular black areas. These appeared due to the evaporation of dextrin binder and as a result of gas confinement during thermal processing. The appearance of pores could affect the thermal conductivity, brittleness, and corrosion resistance of the composite. However, this is not further discussed since the focus of this study is on the general microstructural evolution of the material and its influence on microhardness. All samples were coated by a thin carbon layer in order to improve their conductivity, which made analysis of the C content inaccurate. Despite that, the measured amount of C in a specific area can indicate if it is high or low.

Figure 5 shows the microstructures of samples containing WC_1 powder. Samples with only 10 wt.% addition of WC_1 powder revealed the formation of γ-Ni/intermetallic eutectic in the form of characteristic structures, as marked in the images. Additionally, the WC_1_10_30 sample exhibited the presence of small NbC precipitates. Increasing WC content to 20 wt.% resulted in the formation of large precipitates which showed an increased amount of Cr, Mo, and W. This indicates that the formation of various secondary carbides was possible due to the higher amount of C introduced to the system. While 10 wt.% and 20 wt.% WC_1 samples were mostly similar despite different heating rates, in the materials with 30 wt.% WC_1 addition, differences were significant. The WC_1_30_10 sample showed the presence of large blocky precipitates which consisted mostly of Cr, Mo, W, and Nb, together with an increased content of C. Its formation was possible due to the dissolution of WC_1 particles in liquid alloy, which was followed by the formation of secondary C-rich phases around the remaining WC particles. The presented image shows that the lower heating rate allowed for the extensive formation of secondary phases in the form of fully developed structures. On the contrary, the WC_1_30_30 sample exhibited a large amount of typical fine eutectic structures, together with needle-like precipitates. This was the result of the higher heating rate, which prevented the grain growth of secondary phases particles.

Samples with the addition of WC_2 powder are shown in Figure 6. Materials obtained with a heating rate of 10 °C/min were similar to the previously shown WC_1 samples. Increasing WC content caused the formation of blocky secondary carbides. However, the microstructure of the WC_2_30_10 sample appeared to be much finer than WC_1_30_10 one. This occurred because WC_2 powder did not dissolve completely in liquid Ni. Instead, the surface of introduced WC_2 grains dissolved partially, followed by the additive formation of secondary phases at the WC/alloy boundary, which was the reason for the much smaller sizes of these precipitates. Analysis of WC_2_10_30 and WC_2_20_30 samples revealed black ring structures which were confirmed to have increased content of Cr and C by EDS. Cr carbide formation occurred because of the dissolution of WC introduced onto the Inconel 625 particle surface. Because of the tendency of W to diffuse into the alloy, C was left in the intergranular spaces. It then reacted with Cr originating from the alloy due to its microsegregation. The behavior of these elements was previously analyzed [27] and confirmed in the case of the Inconel 625–WC system [28,29], which agrees with the data presented in Figure 1. This is based on partition coefficient k, defined by the relationship between the selected element concentration in the dendrite core or cell and its average concentration in the analyzed area. It is strongly dependent on the atomic size of selected elements in comparison to base metal matrix atoms.

(3) $$k=\frac{C_{core}}{C_{0}}.$$

This coefficient is determined empirically for different types of alloys. For Ni-based alloys, its values are as follows: kMo ≈ 0.85; kNb ≈ 0.60; kCr ≈ 1.02; kW ≈ 1.12. Results obtained from SEM/EDS analysis revealed an increased amount of Mo and Nb in precipitates. Cr and W were more uniformly distributed throughout the analyzed areas. Because their atomic sizes are closer to Ni, both Cr and W can substitute Ni atoms in the crystal lattice of a metal. The comparison between WC_2_30_10 and WC_2_30_30 samples shows that the higher heating rate of 30 °C/min did not allow the complete formation of blocky structures of secondary carbides, where few eutectic structures were observed. 

**Figure 7:**
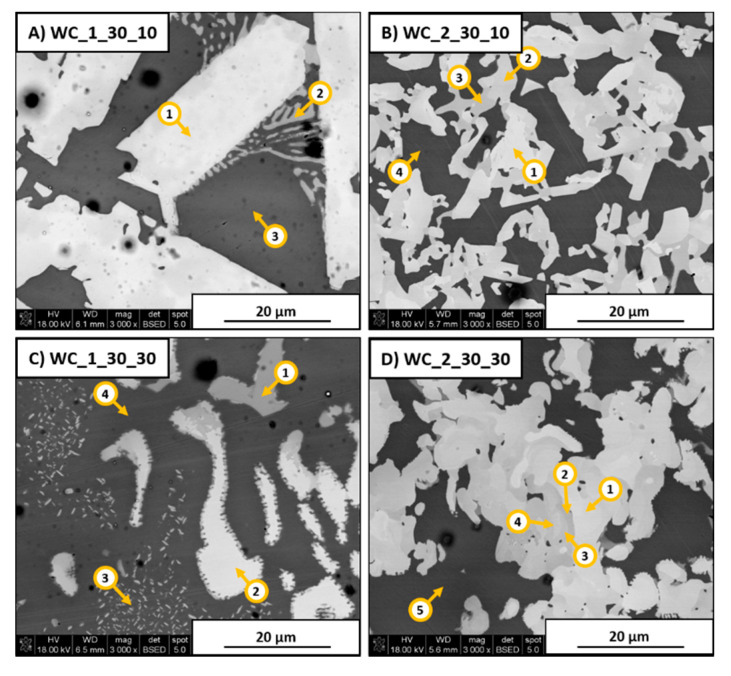
SEM images of Inconel 625–WC 30 wt.% samples with marked areas subjected to energy-dispersive X-ray spectroscopy (EDS) analysis.

It was previously confirmed that laser additive manufacturing methods are characterized by a large amount of heat delivered to powder material, which leads to rapid temperature increase [29]. As seen in Figure 7, major changes were observed between samples obtained with lower (Figure 7A,B) and higher heating rates (Figure 7C,D). The complete SEM/EDS area analysis results are included in Table 4.

As shown in Figure 7A, the WC_1_30_10 sample exhibited the formation of a high number of large white blocky structures in the material. EDS analysis revealed that these structures consisted mostly of W and C with some amount of Ni, Cr, and Mo reported. A very small quantity of γ-Ni/intermetallic eutectic was observed. This indicates the dissolution and recrystallization of large W-rich carbides during solidification. The eutectic had an increased level of Cr and W, most likely due to dissolution of WC in liquid metal. The WC_2_30_10 sample presented in Figure 7B exhibited a different appearance in comparison to the WC_1_30_10 sample. The formation of secondary phases in the form of large grains with different contents of W and Cr as main elements was revealed. No eutectic structures were observed in this material. Faster heating of the WC_1_30_30 sample (Figure 7C) showed that formation of recrystallized WC carbides was not fully completed due to limited grain growth time. W- and Cr-rich white precipitates did not exhibit the regular blocky appearance visible in the material subjected to a lower heating rate of 10 °C/min (Figure 7A). On the contrary, the presence of needle-like eutectic clusters was revealed, as marked by point 3 in Figure 7C. The SEM/EDS analysis revealed the slightly increased content of W and Cr in this area. This indicates the mass nucleation of secondary phases due to faster speed of thermal processing. Elemental concentration analysis at point 1 confirmed the formation of NbC, which was possible due to the high content of carbon derived from WC. SEM investigation of the WC_2_30_30 sample revealed an irregular shape of white and light-gray phases present in this material, as presented in Figure 7D. No appearance of typical eutectic structures was reported. The faster heating rate limited grain growth; thus, it was still possible to obtain elongated precipitates of secondary phases. SEM/EDS point analysis showed that most of these phases contain increased amounts of Cr, Mo, and W.

**Figure 8:**
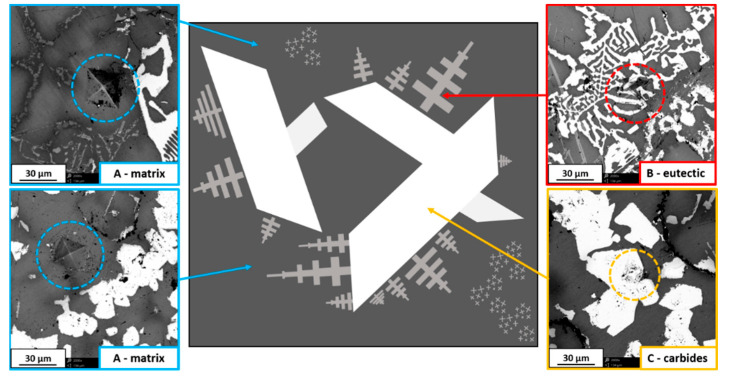
Schematic representation of observed phases with additional SEM images showing typical appearance of hardness test indents in different phases.

**Figure 9:**
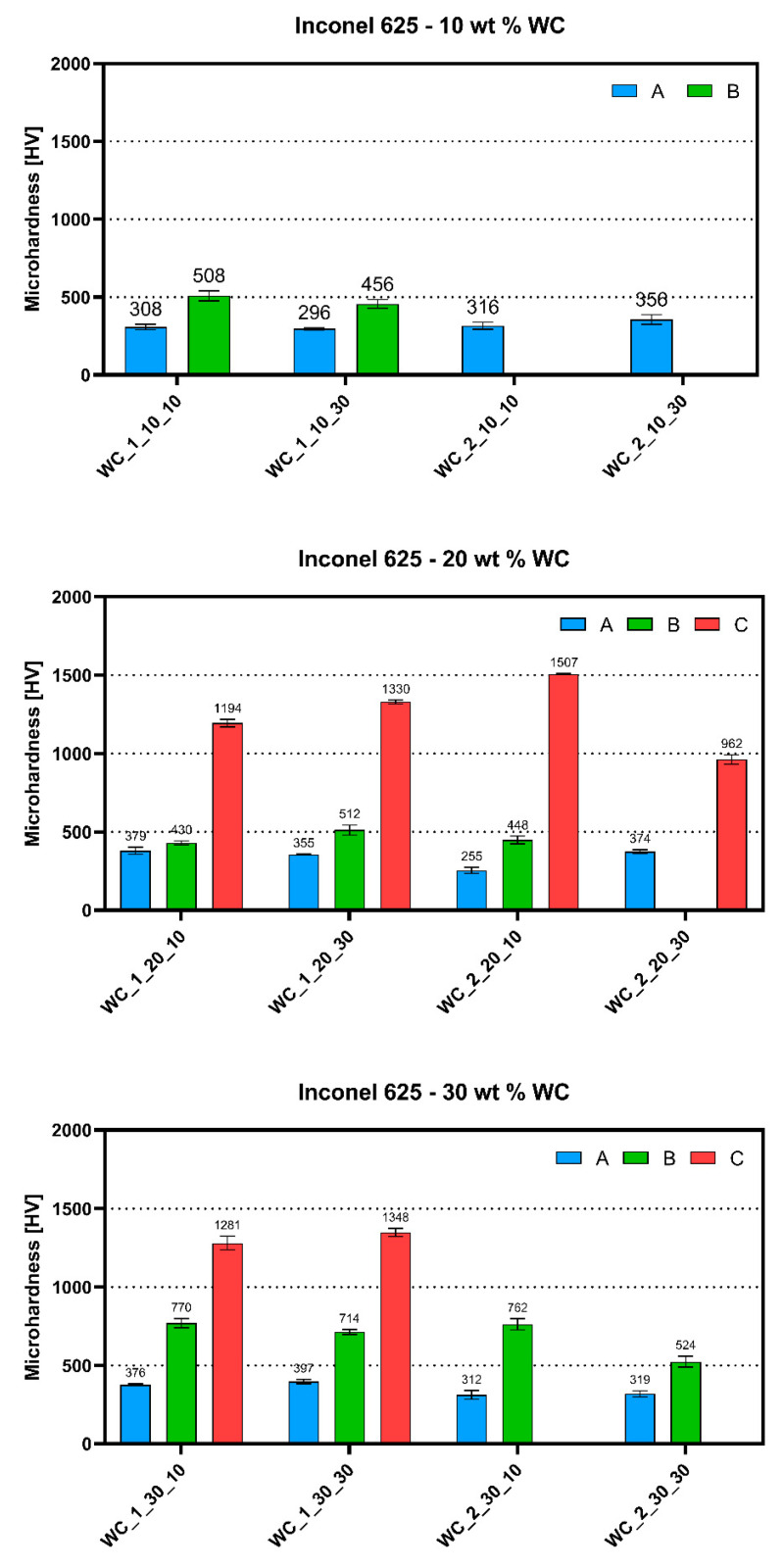
Average microhardness of different phases in DTA-obtained Inconel 625–WC samples measured under a load of 200 g for 15 s where A represents the matrix, B represents the eutectic structure, and C represents carbides.

Microhardness analysis was performed on all DTA-prepared samples in order to investigate differences between structures. The graphs show the maxima of three distinct phases, where “phase A” was the Ni-based matrix of the material, “phase B” was observed as fine irregular eutectic structures, and “phase C” had the appearance of large carbide precipitates observed in some samples, as shown in the schematic representation in Figure 8. The results obtained under a load of 200 g for 15 s are presented in Figure 9. As seen in Figure 8, the appearance of indents made by hardness measurements were different depending on the analyzed phase. It has to be mentioned that the microhardness measured in areas with eutectic structures does not provide an exact value because of its fine appearance. However, it gives information that the areas reinforced by these precipitates were characterized by slightly higher microhardness than the matrix.

As seen below, samples with 10 wt.% addition of WC_1 powder allowed us to perform testing of secondary phase precipitates. The matrices of materials exhibited an average microhardness between 308.64 ± 18.45 and 356.01 ± 31.03 HV, which is significantly lower than the precipitate microhardness of 456.23 ± 28.16 HV (WC_1_10_30) or 508.19 ± 31.77 HV (WC_1_10_10). Due to very fine eutectic structures in 10 wt.% WC_2 materials, it was impossible to measure their value using a standard microhardness tester. Heating ratio changes did not significantly affect the microhardness of distinct phases for samples with 10 wt.% WC content. All of the materials with the addition of 20 wt.% WC showed the appearance of coarse blocky carbides. As seen below in Figure 9, the microhardness of these precipitates was a few times higher than that of the matrix and eutectic structures. An average value of about 1194.45 ± 24.28 HV was reported for the WC_1_20_10 sample. This was even higher for the WC_1_20_30 sample with a value of 1330.20 ± 12.02 HV. The WC_2_20_10 sample exhibited the highest measured value of 1507.48 ± 4.37 HV. The microhardness of the metal matrix and fine eutectic structures was similar to values for the samples with 10 wt.% WC. Materials with 30 wt.% of WC addition had a higher microhardness of eutectic structures between 524.40 ± 34.69 HV (WC_2_30_30) and 770.65 ± 29.15 HV (WC_1_30_10). As shown below, the WC_2 samples did not possess two different precipitate phases because of the larger size of WC that was not dissolved completely, in contrast to WC_1 materials.

The collected results show that both eutectic structures and secondary carbides had a much higher microhardness than Ni-based matrix. The different WC particle size and the heating rate affected the formation of precipitates, which consequently changed the microhardness of distinct phases in obtained materials. A significant increase in microhardness values for irregular eutectic precipitates was observed between 20 and 30 wt.% WC addition samples. This indicates that a higher amount of WC promotes the precipitation strengthening of the Inconel 625–WC system.

## 4. Discussion

The presented results show that the microstructure evolution in the Inconel 625–WC composite is difficult to predict because of simultaneous interactions between elements in the system. The introduction of WC in the form of a powder resulted in the formation of different secondary phases rich in C and alloying elements such as Cr, Mo, Nb, and W, introduced in the form of WC. Additionally, the results provide information about the correlation between processing conditions and selected material properties.

The microstructural observations of obtained materials revealed that the addition of finer WC_1 powder (d_WC_1_ = 3.88 µm) tended to dissolve in the liquid Ni-based matrix during heating. Almost all of the obtained materials showed the presence of eutectic structures except for the WC_10_10 sample. In contrast, the coarser WC_2 powder (d_WC_2_ = 6.13 µm) generally did not allow for such an extensive formation of eutectic structures, which confirms that coarser WC particles can be successfully used in order to prevent their complete dissolution during heating. Heating rates and WC content were confirmed to have a significant impact on material microstructure. All samples obtained under the 10 °C/min heating rate were characterized by a more developed microstructure, where the formation of eutectic structures decreased with higher WC addition. In contrast, a heating rate of 30 °C/min promoted the formation of phases with an irregular appearance, such as eutectics and rounded carbides.

SEM/EDS area analysis revealed the microsegregation tendency of Cr, Mo, and Nb in all materials. An increased amount of alloying elements such as Nb, Mo, and Cr was detected in both eutectic structures and secondary carbide precipitates. Microhardness tests revealed that secondary phases had a higher average value than the γ-Ni matrix. This was the result of Cr and Mo depletion in the matrix which led to a decrease in its microhardness. However, this microsegregation allowed the formation of hard but brittle secondary phase precipitates, which was especially observed with about three-fold higher values for fully developed secondary carbides. The possibility of inducing high hardness in specific areas of a material can be beneficial in some applications such as the surface modification of parts exposed to mechanical abrasion.

The obtained results shown that the microstructural development of a material subjected to a rapid heating–cooling process is highly dependent on the type of powder used and the heating conditions. Via modification of these parameters, a material can be designed in order to fulfill its designated role. In the case of complex systems like Inconel 625–WC, the number of interactions between alloying elements makes it difficult to predict how the material will behave under extreme processing conditions such as powder laser additive manufacturing.

## 5. Conclusions

To conclude, all of the analyzed materials exhibited differences in microstructural evolution when subjected to varying heating conditions of DTA.

- Controlled melting of the Inconel 625–WC system allowed investigating how different heating conditions affect the microstructural development of the composite.
- The higher heating rate of 30 °C/min promoted the formation of eutectic structures due to limited time, which prevented the extensive microsegregation of alloying elements that led to the formation of fully developed secondary carbide grains.
- The addition of WC_2 powder obtained via a heating rate of 30 °C/min caused the formation of Cr carbides at the Inconel 625 grain boundaries, which prevented melting of those grains.
- The higher amount of C derived from WC promoted the formation of Cr-, Mo-, and Nb-rich secondary carbides.
- The microhardness of eutectic structures and secondary carbides was higher than that of the Ni-based matrix of the composite.
- The formation of hard secondary carbides resulted in the depletion of strengthening alloying elements (Cr, Mo, Nb) in the Ni-based matrix because of microsegregation.
- Qualitative analysis of secondary phases in the Inconel 625–WC system is challenging due to the number of elements present in material. Additionally, the low amount of some phases causes problems in accurate analysis.

## Figures and Tables

**Table 1:** Chemical composition of Inconel 625 Ni-based alloy.

Ni	Cr	Mo	Nb	Fe	C	Mn	Si	P	S	Al	Ti	Co	Ta	Cu
Min 58.0	20.0–23.0	8.0–10.0	3.15–4.15	Max 5.0	Max 0.1	Max 0.5	Max 0.5	Max 0.015	Max 0.015	Max 0.4	Max 0.4	Max 1.0	Max 0.05	Max 0.5

**Table 2:** Grain size distribution of WC_1 and WC_2 powders. ID—identifier.

Powder ID	d(0.1) (µm)	d(0.5) (µm)	d(0.9) (µm)	Surface-Weighted Mean D (µm)
**WC_1**	1.40	9.35	19.21	3.88
**WC_2**	3.13	8.59	21.63	6.13

**Table 3:** List of samples subjected to differential thermal analysis (DTA) with analysis conditions.

Sample ID	WC Powder ID	WC Grain Size (µm)	WC Content (wt.%)	Heating Rate (°C/min)	Cooling Rate (°C/min)
**Inc_10**	-	-	-	10	200
**Inc_30**	-	-	-	30	200
**WC_1_10_10**	WC_1	3.88	10	10	200
**WC_1_10_30**	WC_1	3.88	10	30	200
**WC_1_20_10**	WC_1	3.88	20	10	200
**WC_1_20_30**	WC_1	3.88	20	30	200
**WC_1_30_10**	WC_1	3.88	30	10	200
**WC_1_30_30**	WC_1	3.88	30	30	200
**WC_2_10_10**	WC_2	6.13	10	10	200
**WC_2_10_30**	WC_2	6.13	10	30	200
**WC_2_20_10**	WC_2	6.13	20	10	200
**WC_2_20_30**	WC_2	6.13	20	30	200
**WC_2_30_10**	WC_2	6.13	30	10	200
**WC_2_30_30**	WC_2	6.13	30	30	200

**Table 4:** SEM/EDS element concentration analysis results for areas marked in Figure 7 for each of samples.

**WC_1_30_10**		**Ni**	**Cr**	**Mo**	**Nb**	**W**	**C**
**Area 1**	**(wt.%)**	18	7	5	1	62	6
	**(at.%)**	23	10	4	1	25	37
**Area 2**	**(wt.%)**	10	27	9	2	42	9
	**(at.%)**	10	29	5	1	13	42
**Area 3**	**(wt.%)**	61	13	3	1	17	6
	**(at.%)**	55	13	1	1	5	24
**WC_2_30_10**		**Ni**	**Cr**	**Mo**	**Nb**	**W**	**C**
**Area 1**	**(wt.%)**	14	11	12	2	43	18
	**(at.%)**	10	9	5	1	10	65
**Area 2**	**(wt.%)**	14	14	12	3	39	18
	**(at.%)**	10	11	5	1	9	64
**Area 3**	**(wt.%)**	2	25	16	5	31	21
	**(at.%)**	1	18	6	2	6	66
**Area 4**	**(wt.%)**	57	13	3	2	13	12
	**(at.%)**	41	10	1	1	3	43
**WC_1_30_30**		**Ni**	**Cr**	**Mo**	**Nb**	**W**	**C**
**Area 1**	**(wt.%)**	3	4	7	51	24	11
	**(at.%)**	3	4	4	30	7	52
**Area 2**	**(wt.%)**	3	15	8	2	65	8
	**(at.%)**	4	20	6	1	25	44
**Area 3**	**(wt.%)**	36	15	3	2	36	7
	**(at.%)**	36	17	2	1	11	32
**Area 4**	**(wt.%)**	73	9	1	1	11	4
	**(at.%)**	68	10	1	1	3	18
**WC_2_30_30**		**Ni**	**Cr**	**Mo**	**Nb**	**W**	**C**
**Area 1**	**(wt.%)**	2	18	15	2	45	18
	**(at.%)**	1	15	7	1	11	66
**Area 2**	**(wt.%)**	2	25	15	1	38	18
	**(at.%)**	1	20	6	1	9	63
**Area 3**	**(wt.%)**	2	24	14	2	40	18
	**(at.%)**	1	20	6	1	9	62
**Area 4**	**(wt.%)**	2	18	16	2	46	17
	**(at.%)**	1	15	7	1	11	64
**Area 5**	**(wt.%)**	63	10	4	1	10	13
	**(at.%)**	44	8	1	1	2	44
